# A SNP-Based Linkage Map Revealed QTLs for Resistance to Early and Late Leaf Spot Diseases in Peanut (*Arachis hypogaea* L.)

**DOI:** 10.3389/fpls.2018.01012

**Published:** 2018-07-10

**Authors:** Suoyi Han, Mei Yuan, Josh P. Clevenger, Chun Li, Austin Hagan, Xinyou Zhang, Charles Chen, Guohao He

**Affiliations:** ^1^Department of Agricultural and Environmental Sciences, Tuskegee University, Tuskegee, AL, United States; ^2^Industrial Crops Research Institute, Henan Academy of Agricultural Sciences, Zhengzhou, China; ^3^Shandong Peanut Research Institute, Qingdao, China; ^4^Center for Applied Genetic Technologies, University of Georgia, Athens, GA, United States; ^5^Department of Crop, Soil and Environmental Sciences, Auburn University, Auburn, AL, United States

**Keywords:** genotyping by sequencing (GBS), resistance gene, single nucleotide polymorphism (SNP), quantitative trait locus (QTL), *Cercospora arachidicola*, *Cercosporidium personatum*

## Abstract

Cultivated peanut (*Arachis hypogaea* L.) is an important oilseed crop that is grown extensively in Africa, Asia and America. The diseases early and late leaf spot severely constrains peanut production worldwide. Because multiple genes control resistance to leaf spot diseases, conventional breeding is a time-consuming approach for pyramiding resistance genes into a single genotype. Marker-assisted selection (MAS) would complement and accelerate conventional breeding once molecular markers tightly associated with the resistance genes are identified. In this study, we have generated a large number of SNPs through genotyping by sequencing (GBS) and constructed a high-resolution map with an average distance of 1.34 cM among 2,753 SNP markers distributed on 20 linkage groups. QTL mapping has revealed that major QTL within a confidence interval could provide an efficient way to detect putative resistance genes. Analysis of the interval sequences has indicated that a major QTL for resistance to late leaf spot anchored by two NBS-LRR resistance genes on chromosome B05. Two major QTLs located on chromosomes A03 and B04 were associated with resistance genes for early leaf spot. Sequences within the confidence interval would facilitate identifying resistance genes and applying marker-assisted selection for resistance.

## Introduction

Early leaf spot (ELS) caused by *Cercospora arachidicola* S. Hori and late leaf spot (LLS) caused by *Cercosporidium personatum* (Berk. and M. A. Curtis) are the most widespread foliar diseases in peanut (*Arachis hypogaea* L.). Epidemics of leaf spot diseases may result in partial to complete defoliation, which can lead to losses of up to 50% of anticipated yields (Branch and Culbreath, 2013). Multiple fungicide applications are required for leaf spot disease control (Smith and Littrell, 1980). However, even using fungicides, susceptible peanut cultivars are subject to pathogen attack when environmental conditions are favorable for disease development and weather conditions interfere with the timely fungicide applications (Branch and Culbreath, 2013). Although epidemics of leaf spot were less severe using strip than conventional tillage, fungicide treatments are still needed (Cantonwine et al., 2007). Moreover, application of some fungicides, such as chlorothalonil to control foliar diseases may increase the severity of Sclerotinia blight, caused by *Sclerotinia minor* (Porter, 1980) and the incidence of Southern stem rot cause by *Sclerotium rolfsii*. Therefore, the development of leaf spot resistant cultivars is desirable and sustainable strategy to control the leaf spot diseases, which could result a reduction in the amount and frequency of fungicide applications needed to control both diseases (Shoba et al., 2012; Branch and Culbreath, 2013).

The identification and availability of resistance sources influences the success of resistance breeding program. Efforts have been made to identify genotypes resistant to early and late spot from germplasm including peanut breeding lines, commercial cultivars, and related wild *Arachis* species. For instance, a large set of germplasm was screened for resistance to leaf spot and results showed that 87% of resistant genotypes belonged to *A. hypogaea* var fastigiata and 13% to var. hypogaea (Subrahmanyam et al., 1989). Other sources of resistance to late leaf spot were found from the U.S. core collection (Holbrook and Anderson, 1995) and in var. vulgaris and var. hypogaea (Mehan et al., 1996). Closely related wild species, such as *A. hoehnei, A. duranensis, A. ipaensis*, and *A. stenosperma* are potential sources of early and late spot resistance (Pande and Rao, 2001). Recently, additional wild species were identified as sources of resistance and would be useful for the introgression of resistant genes into cultivated peanut (Fávero et al., 2009, 2015). Various resources would also provide the potential for pyramiding different resistance genes into a single genotype. In order to avoid the time-consuming process of screening and selection in a segregating population, marker-assisted selection (MAS) would be a useful scheme to fit into a traditional breeding program. The identification and development of genetic markers linked to resistances to leaf spot diseases would accelerate traditional breeding. Therefore, construction of genetic linkage maps and identification of resistance genes or QTLs for leaf spot resistance traits are important for both traditional and molecular breeding. QTLs for early and late spot resistance have been reported in previous studies using different types of genetic markers and different mapping populations in recent years (Gajjar et al., 2014; Zhou et al., 2014; Kolekar et al., 2016; Liang et al., 2017; Pandey et al., 2017).

In this study, objectives were to construct a SNP-based map through genotyping by sequencing (GBS), to reveal QTLs associated with early and late leaf spot resistance, and to further discover resistance genes by analyzing and annotating all genes within the interval of major QTLs. The outcomes would benefit the MAS programs in breeding leaf spot resistant genotypes and facilitate cloning of resistance genes.

## Materials and methods

### Plant materials and phenotyping of leaf spot in the field

A F_9_ RIL population consisting of 192 individual lines derived from a cross of Florida-07 x GP-NC WS 16 was used as a mapping population (Holbrook et al., 2013). Seeds of this population were kindly provided by Dr. Holbrook from the USDA/ARS at Tifton, GA. The parent GP-NC WS 16 (Tallury et al., 2014) was selected from an interspecific hybridization between *A. hypogaea* and wild species *A. cardenasii*, and has multiple disease resistances including early leaf spot (ELS). The parent Florida-07 (Gorbet and Tillman, 2009) has high oleic oil content along with high yield. Although partially resistant to tomato spotted wilt virus (TSWV) and stem rot caused by *Sclerotium rolfsii*, this variety is susceptible to ELS. The population and two parental lines were grown in the fine-loamy and siliceous soil at the Wiregrass Research and Extension Center at Headland, Auburn University, AL (31°22′N, 85°19′W) in 2015 and 2016 for late leaf spot evaluation. For early leaf spot evaluation, all genotypes were grown in the fine-loamy, mixed, and nonacid soil at the E.V. Smith Research Center, Shorter, Auburn University, AL (32°29′N, 85°53′W) in 2016 and 2017. Genotypes were planted in early May of each year using randomized complete block (RCB) design with three replicates. Each plot had two rows of 3 m long and 0.91 m between rows at a seeding rate of 10 seeds m^−1^. Before planting, the field area was cultivated and irrigated with 15 mm of water as needed to ensure adequate moisture for uniform seedling stands. Crop management for all tests was according to best management practices for soil nutrients, herbicides, and insecticide but received no fungicide. The most common symptom of early leaf spot was identified by brown lesions surrounded by a yellow color on the upper side of leaves and the most common symptom of late leaf spot was detected by dark brown lesions showed on the underside of affected leaves. Intensity of early and late leaf spot diseases were separately evaluated using the Florida leaf-spot scoring system (1–10) 1 week before harvest, where 1 = no disease, the most resistant and 10 = plants defoliated or dead, the most susceptible (Chiteka et al., 1988).

Young leaves of 192 RIL lines were collected from field-grown plants and stored at −80°C for DNA isolation. The genomic DNA was extracted using the modified CTAB method (Porebski et al., 1997). Purified DNA was dissolved in TE buffer for subsequent analysis. The quantity and quality of the DNA were measured using the ND 2000.

### DNA sequencing

Genomic DNA was digested with the restriction enzyme *Msl*l. The 150 bp paired end sequencing using Illumina NextSeq 500 V2 was performed in LGC Genomics GmbH, Berlin, Germany. The approach used for Genotyping by sequencing (GBS) is similar to the double-digest restriction site associated DNA sequencing (ddRAD-seq) but with an *in vitro* normalization. Several steps were perfomed for read pre-processing. Brief, all library groups were demultiplexed using the Illumina bcl2fastq 2.17.1.14 software and allowed 1 or 2 mismatches or Ns in the barcode read when the barcode distances between all libraries on the lane allowed for it. Library groups were demultiplexed into samples according to their inline barcodes and verification of restriction site. Sequencing adapter remnants were clipped from all reads. Reads with final length <20 bases were discarded. All adapter clipped Illumina reads were subjected to quality trimming by removing reads containing Ns and trimming reads at 3′-end to get a minimum average Phred quality score of 20 over a window of 10 based. After quality trimmed, raw data were used to identify SNPs using the SWEEP software (Clevenger and Ozias-Akins, 2015).

### SNP identification

Read sequence from each individual was aligned to the *A. duranensis* (A) and *A. ipaensis* (B) genomes separately using BWA with default parameters. For SNP calling, two methods were used: First, SNPs were called between the parents of the RIL population using Samtools mpileup. Resulting SNPs were then filtered using SNP-ML (Korani et al., 2018). The following command for SNP-ML was used - SNP-ML -c 0.7 -i input.vcf -iM peanut_DNA -o outputs. The option sets the stringency higher for accepting SNPs that have a better model fit. The program was designed using neural network machine learning and developing a model using large sets of validated TRUE and FALSE SNPs originating from the Axiom Arachis58K SNP array (Clevenger et al., 2017). The models have been validated at more than 80% accuracy of filtering false SNPs. Second, SNPs were called using all individuals in the population and then subjected to SNP-ML filtering.

Resulting SNP sets for the A and B subgenomes were then called in the population using a custom python procedure that uses Pysam (https://github.com/pysam-developers/pysam) to observe every base covering the SNP site from every read mapping to that site. The genotype calls were filtered as missing if there were <3 reads covering the SNP site in the individual. Because peanut is an allotetraploid, heterozygous genotypes were not called and markers were treated as dominant.

Called genotypes were filtered further for segregation distortion and missing data using custom python scripts. A chi-square test was conducted for a 1:1 segregation ratio. Any marker that had a *p*-value below 0.05 was filtered out. Markers were then filtered out if they contained more than 80% missing data.

### Genetic Map construction

Construction of a linkage map was carried out using JoinMap 4.0 version (Van Ooijen, 2006). The file of MS-Excel containing SNP markers aligned to each chromosome were loaded into JoinMap 4.0. A minimum LOD score of 2.0 and maximum recombination fraction of 0.5 were set as threshold values. The navigation panel showed linkage groups through a function of *tree view*. The recombination fraction was converted into map distances in centi-Morgans (cM) using Kosambi's function (Kosambi, 1944).

### QTL analysis

All necessary computations for QTL mapping and estimation of additive effects were performed using the WinQTLCartgrapher software 2.5. Composite interval mapping (CIM) with cofactors selection step was used for the detection and mapping of QTLs (Zeng, 1994). The genotypic data of SNP markers and the mean phenotypic data of replications in the parents and RIL lines were used for QTL analysis. A 2.0 cM window size was used for the genome scan. The threshold LOD score was estimated empirically using 1,000 permutations at 0.05 to declare significance for all the traits evaluated in the study. The presence of a putative QTL was declared if the LOD threshold was over 3.0 for the traits. The QTLs showing phenotypic variance explained (PVE) >10% were considered as a major QTL. MapChart 2.3 was used to draw a final genetic map including all QTLs for better visualization (https://www.wur.nl/en/show/Mapchart.htm).

## Results

### Phenotypic evaluation

**Figure 1:**
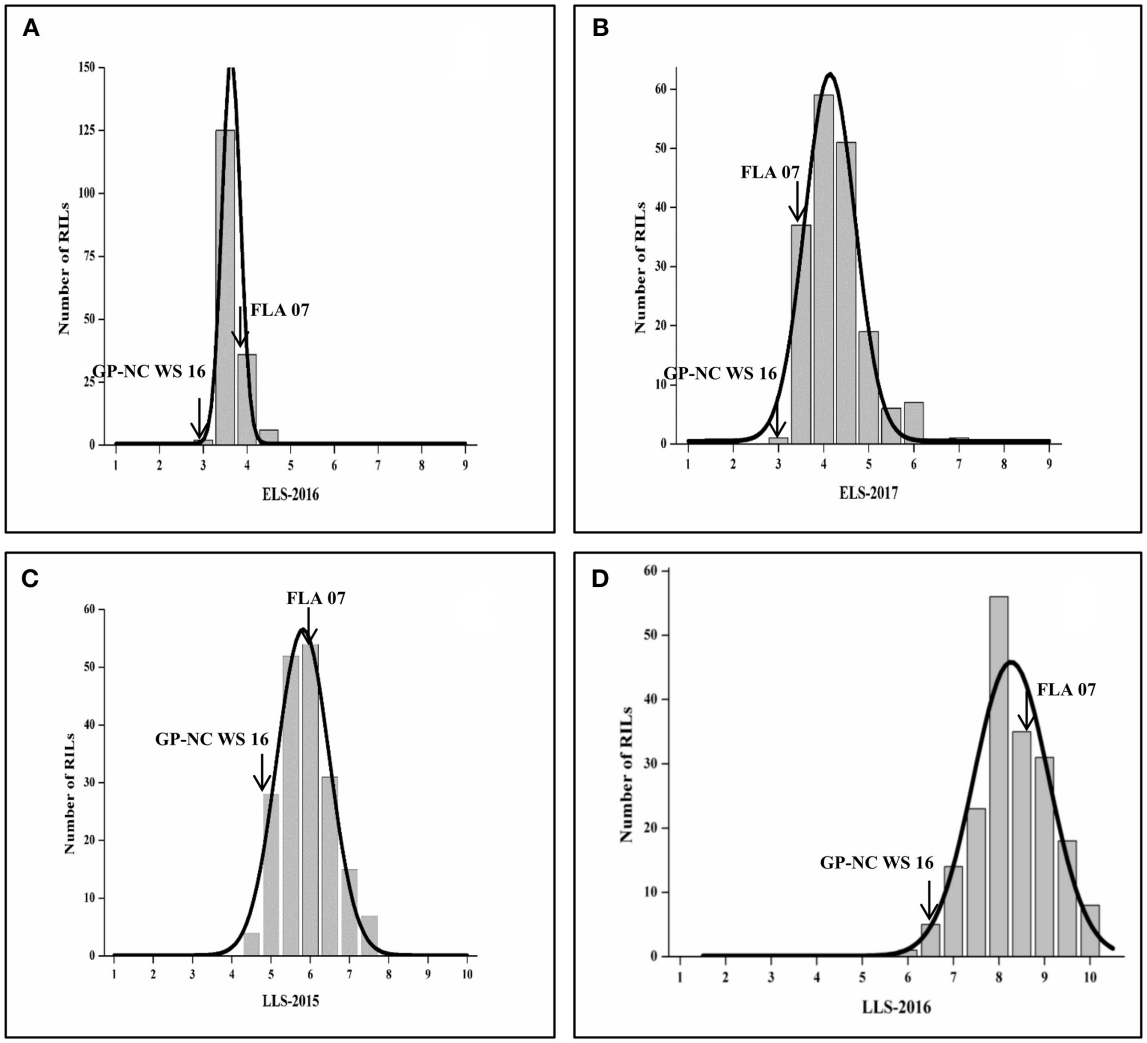
Frequency of ELS and LLS disease scores in F9 RIL lines in different locations and years. **(A,B)** ELS at Shorter, AL in 2016 and 2017, respectively; **(C,D)** LLS at Headland, AL in 2015 and 2016, respectively.

A set of 192 RILs was phenotyped for ELS in 2016 and 2017 and LLS in 2015 and 2016. Phenotypic data displayed near normal distributions for both disease evaluations in both years. In general, they were more consistent for late leaf spot than for early leaf spot from year to year (Figure 1). The range of the rating for LLS was 4.0 to 8.0 in 2015 compared with 5.8 to 9.8 in 2016. For ELS, the ranges of ratings were shifted from 2 to 5 in 2016 to 3 to 6 in 2017, but the frequency of distribution was skewed to more susceptible in 2017. The ratings for ELS in 2016 have a small standard deviation (α = 0.43) vs. (α = 0.67) in 2017, indicating less variability of ELS evaluations. The test of ANOVA showed a significant difference (p < 0.0001) in G x E interaction for both diseases, indicating diseases epidemic were affected by environment (Table S1). Two parental lines as resistant and susceptible checks performed as expected throughout all the tests.

### Sequencing data

Sequencing has generated 337,310,705 read pairs and resulted in average 1.5 million of read pairs per sample. Raw data have been submitted to GenBank and the SRA accession number is SRA132381 (http://www.ncbi.nlm.nih.gov/sra/SRA132381).

After SNP calling and removed heterozygous alleles, total of homozygous 3,672 SNPs were identified and showed polymorphism between two parental genotypes, from which 2,540 were physically aligned to the A genome and 1,051 aligned to B genome, and the remaining 81 were ambiguous and aligned to scaffolds.

### Linkage map analysis

**Figure 2:**
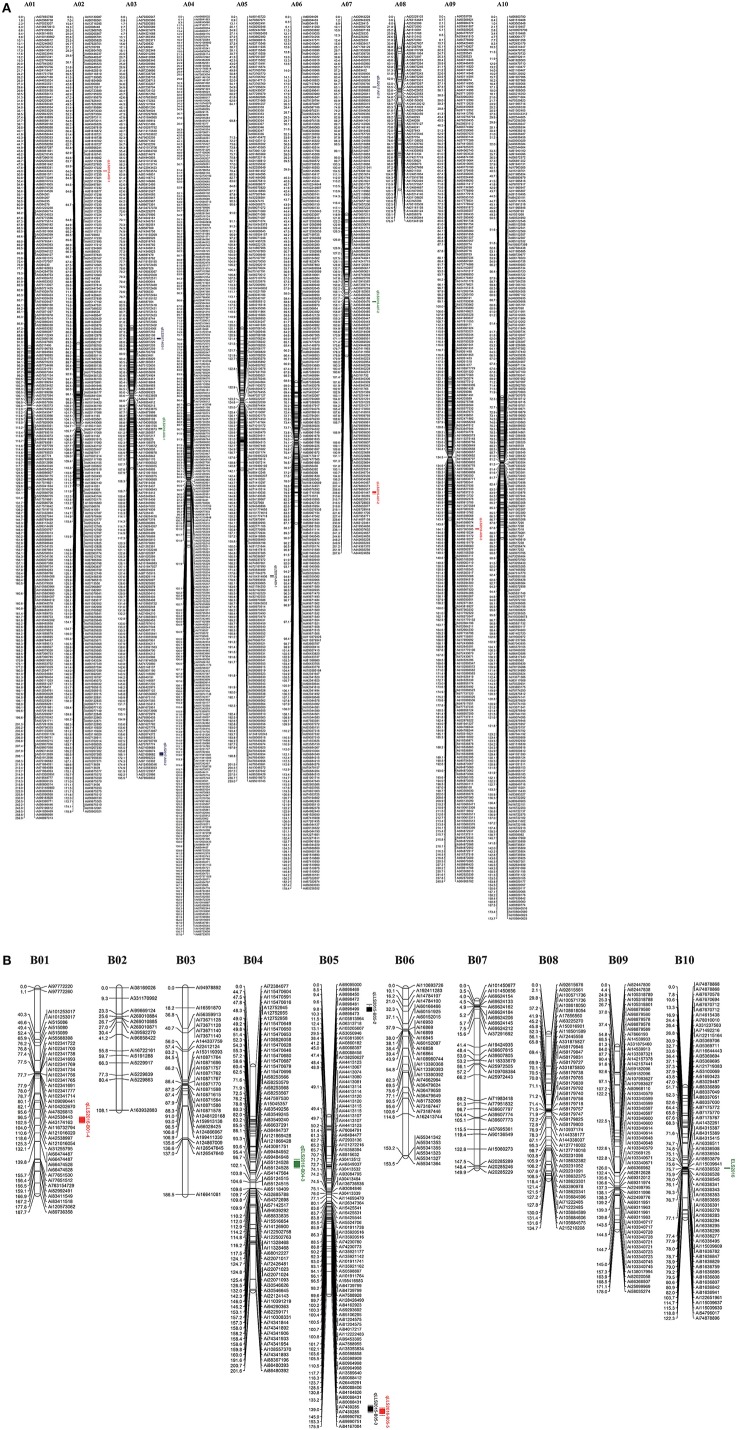
Genetic linkage map of the F_9_ RIL population derived from the cross of Florida 07 and GP-NC WS 16. Left of each group is map distance (cM) and right is the name of marker with physical distance (bp). **(A)** A genome map with 9 QTLs for resistance to early and late leaf spot diseases; **(B)** six QTLs for resistance to early and late leaf sport diseases identified in the B genome map.

After segregation distortion and missing data were filtered, 184 RILs with 3,672 SNP markers were used for the construction of a genetic linkage map. All SNP markers physically mapped to each chromosome were separately used to form each linkage group using JoinMap 4.0 based on the recombination frequencies that occurred in this population. The remaining 81 physically unmapped markers were added into each linkage group as determined by JoinMap 4.0. As a result, 2,266 SNP markers were mapped in A chromosomes and 487 mapped to B chromosomes. A total of 2,753 markers were assigned into 20 linkage groups spanning a genetic distance of 3695.4 cM with an average marker interval of 1.34 cM (Figure 2) with the remaining 919 markers unmapped. The number of markers mapped in each linkage group ranged from 14 to 318 and the lengths of linkage groups ranging from 108.1 to 267.6 cM (Table 1).

**Table 1:** Distribution of SNP markers and linkage group length in each chromosome.

**Chromosomes**	**Number of markers aligned**	**Number of markers mapped**	**Linkage group length (cM)**
A01	258	240	259.86
A02	265	238	187.83
A03	264	228	195.53
A04	342	318	177.63
A05	270	229	239.02
A06	290	261	178.43
A07	226	161	267.61
A08	145	62	176.85
A09	280	259	240.76
A10	302	270	173.72
B01	101	41	187.65
B02	123	14	108.10
B03	113	30	186.54
B04	148	73	201.64
B05	151	92	175.89
B06	141	32	153.52
B07	102	28	149.90
B08	152	48	134.73
B09	125	63	177.97
B10	145	66	122.26
Total	3,943	2,753	3,695.43

### QTLs for resistances to ELS and LLS disease

QTL analysis of genotyping data with phenotyping data in 184 RIL lines was carried out for ELS and LLS disease resistances using WinQTLCart version 2.5. As a result, 15 genomic regions were identified associated with resistance to leaf spot diseases in the Florida-07 x SPT06-06 population within three years. For LLS, eight QTLs were identified in six linkage groups between two years, among which 2 major QTLs (*qLLS2015-B05-2* and *qLLS2016-B05*) were mapped on the same position in the B05 group with 11.64 and 16.6% PVE in two years, respectively (Table 2). Although another QTL (*qLLS2015-B05-1*) was mapped on the opposite end of the same linkage group, its physical position was nearby the region of these two major QTLs (Figure 2). Two major QTLs for ELS were located on different linkage groups, A03 and B04 accounted for 11.67 and 10.63% PVE in 2016 and 2017, respectively. The remaining QTLs exhibited < 10% PVE. To validate these identified QTLs in this study, three major QTLs with PVE >10% (*qLLS2016*-*B05, qELS2017*-*A03*, and *qELS2016*-*B04*) were selected to investigate the sequences within their intervals. QTL mapping on this high-density map generated narrow QTL intervals with sequence length ranging from 1.5 Mbp (qELS2017-*A03*), 2.4 Mbp (*qLLS2016*-*B05*), to 12.5 Mbp (*qELS2016*-*B04*), which facilitates us to scan intervals for resistance gene content. Although these three intervals were still quite wide, they could be downloaded from reference genomes (https://peanutbase.org) based on their physical locations. The FGENESH program (http://linux1.softberry.com/berry.phtml?topic=fgenesh&group=help&subgroup=gfind) was used to predict candidate CDS and protein within these interval sequences. The program has generated 436, 670, and 4641 models as putative genes in QTL intervals of A03, B05, and B04, respectively. All models were subjected to annotation by the Blast2GO program. After removing those models that were unable to blast due to short sequence or no hit, and the models of uncharacterized proteins, 130, 320, and 1,076 models were annotated. As expected, *qLLS2016*-*B05* sequences contained two NBS-LRR resistance genes and one pathogenesis-related (PR) protein gene, as well as several genes coding for regulators of gene expression, such as transcription factors, DNA binding domains, and auxin responsive proteins. The *qELS2017*-*A03* possess two homologs of TMV resistance protein N and *qELS2016*-*B04* contains one PR gene and one homolog of TMV resistance protein N. Each of these two major QTL regions also contain several leucine-rich repeat (LRR) domains. The DNA sequences of these two NBS-LRR and two TMV resistance genes were listed in Table S2.

**Table 2:** QTLs for resistances to ELS and LLS diseases in different locations and years.

**Trait**	**QTL name**	**Linkage group**	**PVE (%)**	**Confidence interval (cM)**	**Additive effect**
LLS-2015	*qLLS2015-A05*	A05	5.26	177.7–178.7	0.26
	*qLLS2015-B05-1*	B05	5.03	3.2–10.3	0.26
	*qLLS2015-B05-2*	B05	11.64	136.1–142.3	0.30
LLS-2016	*qLLS2016-A02*	A02	7.36	63.9–64.3	0.34
	*qLLS2016-A07*	A07	6.53	208.2–210.8	0.32
	*qLLS2016-A09*	A09	6.95	143.8–144.6	0.32
	*qLLS2016-B01*	B01	9.86	97.9–102.9	0.36
	*qLLS2016-B05*	B05	16.60	137.2–143.0	0.39
ELS-2016	*qELS2016-A03*	A03	7.59	99.4–100.4	0.25
	*qELS2016-A07*	A07	6.40	157.8–158.8	0.25
	*qELS2016-B04*	B04	10.63	94.8–102.0	0.32
	*qELS2016-B10*	B10	8.15	75.6–76.0	0.25
ELS-2017	*qELS2017-A03-1*	A03	7.00	87.6–88.7	0.26
	*qELS2017-A03-2*	A03	11.67	163.7–168.4	0.28
	*qELS2017-A07*	A07	4.93	64.8–65.6	0.25

## Discussion

Genetic linkage maps of the cultivated peanut were not available until the first decade of the twenty first century after SSR markers were identified (Hopkins et al., 1999; He et al., 2003; Ferguson et al., 2004; Luo et al., 2005; Proite et al., 2007; Cuc et al., 2008; Gautami et al., 2009; Guo et al., 2009; Koilkonda et al., 2011; Zhao et al., 2012). Although more than one thousand polymorphic SSR markers have been developed, the number of SSR markers mapped in the populations derived from bi-parental crosses was limited to 300-400 due to the narrow genetic base in this crop (Hong et al., 2008; Varshney et al., 2009; Qin et al., 2011; Wang et al., 2012). Without a high-resolution linkage map, it is difficult to precisely identify QTLs associated with traits of interest. In recent years, a large number of single nucleotide polymorphism (SNP) markers was detected via GBS as the price of next generation sequencing (NGS) has declined. Abundant SNPs distributed along the entire genome would be an excellent type of marker to construct a high-resolution linkage map. Two SNP-based linkage maps in the cultivated peanut were currently developed with 1,621 SNP markers in the population of Zhonghua 5 × ICGV86699 (Zhou et al., 2014) and 1,211 SNP markers in the population of Tamrun OL07 × Tx964117 (Liang et al., 2017). Both studies have used ddRAD-seq protocol with two different restriction enzymes, *Sac*I and *Mse*I vs. *Pst*I and *MluC*I, for the library preparation in two populations, respectively. In the current study, we have utilized the GBS technique similar to ddRAD-seq with one restriction enzyme *Msl*I that resulted in 2,753 mapped SNP markers. Both approaches allow high-throughput sequencing and generate a large amount of raw data that are used for SNP identification. This is the key step to discover informative SNPs from the raw dataset. Several bioinformatics tools for SNP discovery have resulted in different number of SNPs. Zhou et al. (2014) used SOAP software (Li et al., 2009) for SNP calling while Liang et al. (2017) used the Bowtie2 method (Langmead and Salzberg, 2012) for alignment to reference genome. We used the SWEEP software (Clevenger and Ozias-Akins, 2015) along with SNP-ML (Korani et al., 2018) for SNP identification. All reads were aligned to the reference genomes (http://peanutbase.org) and SNPs were called using Samtools mpileup and SNP-ML filtering. The number of heterozygous alleles were greater than homozygous alleles due to the allotetraploid feature in peanut and similarity between the two subgenomes. After eliminating abundant heterozygous alleles, homozygous alleles were used for constructing a map based on their physical distances. The physical map consisted of 3,672 SNP markers that formed the basis for genetic linkage map construction based on the recombination that occurred in this population. The genetic linkage map included 2,753 SNP markers with 919 unmapped. Each mapped marker with its physical distance facilitates tracking down its physical location in the genome. The approach we used in this study is useful in the peanut mapping project by using GBS technology.

Complex inheritance pattern of ELS and LLS with multiple genes conferring resistance and interaction between the two diseases make phenotypic selection less effective (Tiwari et al., 1984; Green and Wynne, 1986; Kolekar et al., 2016; Zongo et al., 2017). The breeding efficiency for disease resistance can be enhanced by utilizing a MAS approach where DNA markers are associated with resistant traits. Several studies have demonstrated that DNA markers and QTLs were identified linked to resistances for ELS and LLS diseases (Gajjar et al., 2014; Kolekar et al., 2016; Zhou et al., 2016; Liang et al., 2017; Pandey et al., 2017; Zongo et al., 2017). However, the marker-trait association identified from these studies cannot be comparable because different types of markers were used and the linked markers or QTLs were located on different linkage groups without their physical positions. Additionally, resistance sources might be different because mapping populations derived from different bi-parental crosses that may possess different resistance genes were used. For instance, one major QTL was found on B05 in our study, while several QTLs resided on B06 in the study of Zhou et al. (2016) and two major QTLs were on the A06 and A05 identified with SSR markers by Pandey et al. (2017). Although the former two studies have identified QTLs using the same type of markers, SNP markers, the mapping populations used for map construction in the three studies were different. Similar comparison of QTLs for ELS, the major QTLs for ELS identified with SNP markers in our study were located on the A03 and B04 chromosomes while major QTLs identified by SSR markers were mapped on A05, A06, A09, B03, and B07 groups in the study of Zongo et al. (2017). Because less polymorphic SSR markers were available in peanut compared to other crops, QTL mapping using SSR markers may result in a wide interval. Thus, these markers flanking a QTL may not be tightly linked to a resistance gene and that may lead to recombination occurring between a marker and the gene, reducing the reliability and usefulness of the marker.

The fine mapping of QTLs in this study could facilitate the isolation of putative resistance genes by directly analyzing sequences within confidence intervals. These putative genes would provide a potential for MAS approach in peanut breeding. All defined candidate resistance genes need to be further explored for their functional studies. Cloning of these genes underlying QTLs would unravel the genetics behind resistance to leaf spot diseases in peanut.

The accuracy of QTL mapping can be significantly affected by phenotyping and genotyping data. Genetic distances among markers are dependent on chromosome recombination that requires a large population size and accurate phenotypic evaluation. In this study, two major QTLs (*qLLS2015*-B05-2 and *qLLS2016*-B05) for LLS were detected on the B05 chromosome between two years' phenotypic evaluation and their physical locations overlapped. A resistance gene located within this narrow region would be reliable and useful in molecular breeding of peanut. However, two major QTLs (*qELS2016*-B04 and *qELS2017-*A03-2) for ELS were found on different chromosomes between two years' phenotypic evaluation. The distribution of most ELS phenotypic scores was significantly toward susceptible even beyond both parental scores in 2017. As stated above, the ratings of ELS in 2016 had less variation (α = 0.43 vs. 0.67) than in 2017. Late summer weather patterns likely contributed to differing early leaf spot intensity observed in 2016 and 2017. For 2016 late summer weather was dry compared with 2017 when hurricane Irma brought flooding rains in early September to Shorter, AL which greatly intensified ELS intensity as compared with the previous year. Therefore, multiple years and multiple locations for disease evaluations of ELS are required.

## Conclusion

Identification of resistance genes is not only useful in gene transformation by biotechnology but also benefit in marker-assisted selection for introgression in conventional plant breeding. This study provided putative resistance genes to early and late leaf spot in peanut. By construction of a SNP-based genetic map using genotyping by sequencing approach, major QTLs were identified associated with the resistances to early and late leaf spots. Analysis and annotation of genes within confidence intervals of major QTLs, two NBS-LRR resistance genes for LLS and two homologs of TMV resistance protein N for ELS were revealed. The identified genomic regions and putative resistance genes to early and late leaf spots will be further studied for application of the MAS approach in peanut breeding.

## Author contributions

SH, MY, CL, and XZ: conducted the experiments and data analysis; JC: performed SNP discovery; CC and AH: prepared plant materials and phenotyping; GH and CC: conceived and designed the experiments; GH, SH, JC, and CC: developed the manuscript draft and all authors contributed critically to the draft and gave final approval for publication.

### Conflict of interest statement

The authors declare that the research was conducted in the absence of any commercial or financial relationships that could be construed as a potential conflict of interest.

## Supplementary material

Table S1 ANOVA of phenotypic data for LLS in 2015 and 2016 and for ELS in 2016 and 2017.

Table S2 List of resistance genes identified on B05 for LLS and on A03 and B04 for ELS.
