# Supplementary material for: A SNP-Based Linkage Map Revealed QTLs for Resistance to Early and Late Leaf Spot Diseases in Peanut (Arachis hypogaea L.)
Source: Front Plant Sci. 2018 Jul 10;9:1012. doi: 10.3389/fpls.2018.01012 (PMC6048419; doi:10.3389/fpls.2018.01012)
Supplement: Table S1 — ANOVA of phenotypic data for LLS in 2015 and 2016 and for ELS in 2016 and 2017. [file Table_1.DOCX]

Table S1. ANOVA tables

I. ANOVA of late leaf spot rating scores in 2015 and 2016

| **Source** | **DF** | **SS** | **MS** | **F Value** | **Pr > F** |
| --- | --- | --- | --- | --- | --- |
| Line | 194 | 567.049 | 2.923 | 12.27 | < 0.0001 |
| Year | 1 | 1646.402 | 1646.402 | 6910.49 | < 0.0001 |
| Line X year | 190 | 110.918 | 0.584 | 2.45 | < 0.0001 |
| Error | 772 | 183.927 | 0.238 |  |  |
| Corrected Total | 1157 | 2508.296 |  |  |  |

II. ANOVA of early leaf spot rating scores in 2016 and 2017

| **Source** | **DF** | **SS** | **MS** | **F Value** | **Pr > F** |
| --- | --- | --- | --- | --- | --- |
| Line | 194 | 104.500 | 0.539 | 2.23 | < 0.0001 |
| Year | 1 | 60.477 | 60.477 | 249.99 | < 0.0001 |
| Line X year | 170 | 62.373 | 0.367 | 1.52 | 0.0002 |
| Error | 557 | 134.750 | 0.242 |  |  |
| Corrected Total | 922 | 362.100 |  |  |  |
