# Supplementary material for: A SNP-Based Linkage Map Revealed QTLs for Resistance to Early and Late Leaf Spot Diseases in Peanut (Arachis hypogaea L.)
Source: Front Plant Sci. 2018 Jul 10;9:1012. doi: 10.3389/fpls.2018.01012 (PMC6048419; doi:10.3389/fpls.2018.01012)
Supplement: Table S2 — List of resistance genes identified on B05 for LLS and on A03 and B04 for ELS. [file Table_2.DOCX]

R genes identified on B05 for LLS and on A03 and B04 for ELS.

>B05:8860908..8866178

AGAGCATGAATTACTCCACCAGACACGCATAATAACATCATCAACAACCTGAAACAGAAA

AGGATAGAGGAAATCAGGTTACCAAGTTGAAAAAGCAAAAATTGACCATGAAACTTCTAT

TATTAAATATGTATAATATACAACCTCTTTGTCAAAGGCAACACTAAAAAAAAAAAAAAG

GCAGCAAAATAAGCATTTATAACACCTAATCATCTATAAATTACATGTGATAAAGCTCTA

CATAGGGTATAATCATGTCTACAGTTAACGAAGACAAAAATGCAACCAAGACTCCGGGAG

TATAAGCCAACTCAGAATCATCACATAAATGCTTGATTTAGGATAAAGTATTATTTTAAC

TTTAGATGAAGTTTTACTTTTGTCGTTAATGTTTGGACCGCCTAAATGCACAAATAAAAA

CTATGCAATGTAGTTATTGACTTATTGTTGCACATATATTCGGCCAAATAGGGTGATTAT

TGATTACGTAAATAATAAGAAAAAAAGAGTTACAATACTTACTATTTTGAATTCACTCTT

AATTTACAGGTGGACTAGAAGTAGGGGTGGCAACACTACCCGAACCCGCGGGTACCCACC

CCGCCCCTACCCGCTCGGNGGTCAAAGAATACAGTTTCATATTTTGACAAGGAGATAATA

CTCAATTTGGTCCGTGAACTTGTATGCAAGCCACAATTTAGTCCCTAAAATTTTAATTGC

CTTTATTTAGTCCTCAAACTTTATAAACATAACTCATATTAGTCCTTGAAACAATTTTCA

ACGTACAAACATTAACGGAACACTGTTGTGAACAGCCGGATGCCACACTAAACCTTGTAA

AATGATATCATTTTAGTTTTGGGATTCAAATAACCCAAAAACACCATTTACCCCAAAACA

ACATTGTAAATGGTGTTTATTAAAATTTTACCTAACAAAATAAGTGATATGATACCGTTT

TTGAACTATTTAAATGCCAAAACCAAAACTACATCATTTTACAAGTTTTAATGTGACATC

TGATAGTCTATAACAGCGCTCTATTAATGTTTTTATACTAAAAATCGTCTCAGGGACTAA

TATGAGGCACGTTAATAAACTTTGGGGACTAAATAGAGACAATTGAAACCTCAGGGACTA

AATTGAGACTCGCGTGTAAGTTCAGGGATCAAATTGAATATTAACTCTTTGACATTCTTT

AAGTTTGTTACTAATAAGATAATAAATAAATAATTCTACAGATAAAAAATATAAAATAGT

TTATAATGGTACTCTTTGAATTTTTAATGACTTAAAATATTCAATAATTGAATCAGAACT

AAACTTTTCAACAATTTCTTTCTCAATGTAAATAACCAAACTATCCGCAAGAAAGTCGTC

CTCCATCTTGTTTCTTAACCTTGTCTTTATTATTTTCATTGCTGAAAATGATTGCTCTGT

GGTAGCTGTAGAAACTGAAAGAGTTAATACAGACGAATCAATTTATCAATCAAGTAATAC

ACTTTTGATTTTTTTGTTTCTGCTAAACATCTACACAGTTCATGAATAGTTGACAAATTC

TTCATTTTTAGATGCTGACGAACATCAAGAATAAAATGCTGAAGTTGAAAAGGCAAATTA

ATCTTCTCTTATTCAATAAAGTTTTCGGGATAGTAGCTGTCAACAAGAATAGAAATCTTA

GCAATGCCAAAATTTTTATAAGCATCCTTAGACATGAGAGTAGAGCTCAAACTTAATAGA

TCCATTGCTTGATTATTAAATTAAAGCATCTAAGCCAACTAAACTATTTTTTATTTTTTG

AAAAACTACTACTAATTTTTTTATAAAAAATTTGGGGTAGCCATGCCACTGTCAGCAACG

AACTCCATGAAGCCAATTTAAGCTGATGTCTACTTTAGCTGTCTCTATCTTCAAATTGGG

AGTTGAGGGGACATTTTCCCATAATTGAGCCCTCTCCTCATCACATAACACAAATACTTG

ATGTTTTGCATTCTCAAAGTTCATGACTGAGTATGGCACCTCACTTAGCCCTGAACAACC

CTTCATGTAGAGTTTCTCAAGATTCTGCAGATCTCCAATGTCCTCTGGCAATTTGGATAG

ACTTACACAGTCAGATATGTCGAGACAGCGCAGGTTACACAGTCGTCCAATCCCATCTGG

CATCTCTTCCAAATCGGAGCATGAACTTAGCCTTAGCACTTCCAAATTCTTCAGATTTGC

TGTGTCTTTAGGAAGTGCTGAAAGCTTGTGACAGTTGGTGATGCTAAGCTTCTTTAGTGG

GGTAATGTTGCATACCCCCTCTGGCAATTTCTCCAGATCTTTGCAATAGTCAATGCTCAA

CTCCACTAGATTTGGCATGGATTCTGAGATTGGGATAGAACTGTTCCCAAAAGCCTGGCT

TGTGTTGCACATATGAAGCGATAGTTTTTGCAGACTCTTCAGTATGCATAAAGAAGGAAC

TGAAACTTTCTCCAATCTGATTCTTCTCAAGTTAGACAAAGCACTCAGTATCTCAAAATC

TTTTAGTCCAGAAAGATGGAAGCCGTAATTTGTGACTATTAGAACTTTAAGTTTGTTCAT

TTTCTCCATGAACTGTGGCAGGCTGTACTGGCTAGATCGAATGTTTAAGACTATAACCTC

GGCTTCATCAGCTGTCATGTCGCACCAATCCGAAGCAAAATTCTCGTCTGCCCAAGCATC

ATATATGGCATCAAGAACACAAGATTATGCAAGATAAAGTAATTTAGATTTTAGAAGAGA

GGAATTCACAAACCAGTTGATATAGACAATACTCTGGCAGCAACCTTTAGCTGCTTTTGT

TCTATCAATCTTCCAGGTAAGAATGACAGTGACAAAAGGCGGCTGAATATTCCTTGCTGG

TTTTGCCTTACCCACCATTCAGGATGATCATCTCCATTTAGCTCAATTATTAGTTTCTTT

CTCTGTTCAATTTGTTCCTGCTCACTCTGATGGATTGCCAGCTTTCTGAGGAGATCATGT

AGCAAGACAAAGTGGTTATTGTAGTACATGTCTGTGTCCTTTGCAATTTTCCTGATCAAT

TACCATAGTTTAGTCCAGCTATATTATGGAATCAATCTTTACTTGTCAATAAATCAAGCA

TTTTTAGATAACCAGCTACGAAAAGGATAAAGGATGATGTGTACTTTGTTACTATGAGCT

TAATCAAGTTCCTGGTGGTTAAATCATGGATGATGGTCATTGCTTTCCTTCCATTGTCAT

CAAAGTTATACAGTTCTGCCCACATATCAATCAGGATTGCAACAGGGATCCTTTGGTCTT

CAGGAAAAAGGCCTAGATCCATGAAGCACTCCTTCTCACTAACTGAGAACTTATCTTCCA

ACATATCTAAACTTTGTTGGAGCCGCGAAAGCAAATCTGTATTACCCGACTCCAAAATCG

ACTGCTTTTCTAACCATTCTTTCATATCTTGCCACACCTCAAAAGGCTGCTGACACAGTG

ATCCACCAATCACTTCAAGTGCCAATGGTGAACCCTTACAACCTTTCACTATCTGAATTT

CAGTGCAACAAAGCAAAAAATCAGTGAACAAGATCAAACAAAGAGCTTGAAAGAGTTACA

TGGAATACAATACACACATTCAAAGTAGAAAGAAGATAAAGCACCTCATTGACAAGATTA

TCATCTGGCATGTAGGAGCATTTACTATTAAGTTGAGCCGAGTGACGGAAAAGGGACACT

GCATCATCATGATCAAGGGGTTCCAACGGCCATGGGGTGCCAAATCTTTGAAAAGCAACC

CTTGAAGTCACCAAAATCTTATAATCTGGTATCTGAAATCTGAACTTGTCAACAAGTGCC

TCTGAGCTCTGCCAAACATCATCCAGGACTAATAGTATTGGGCTTCTCCCAACAAGCCTC

AACAAAATTCCCAATTTGTTGATGGCGTCTTCATCACTCTGAAACTCGGGCACCTTACAT

CCGCAGTGCTCAAATAATGTCCTTACAATGTTCTTCAAGTTTGGTGTTTCTGAGAAGGTC

ACAAAGAAGATATTCCCCCCGAACTTGCCTAGCCAAACCAAGGTAAATTTCTATAATCAA

GTAGTATCTAGAAGTATTCCAGAATGCATGATATGCATGACCATTTCACTAGACTAAGAA

GAGTAACGGTATATAACTGAAAGCACCTACCTCAATTCTCAAGGCCACTGTAAACCTAAT

GTATCTGTTAATGCATATGCATAGATAATTATGGTAATATGAATAAAAAAGGAGAAAGGA

ATTCTTTTCATATTGAAAAACATCAAAATTTTTACGAGGCTTTCCCAATTGAGATACTGT

AACTAACAAGAATGGACCACATTCCCGAAATAGCTCAAGTATTGAACTTGGTTTTGGTAC

GGTGACGGATACACATGACATTCTGGTATGCAAATAAATTGGTATGTGGTACTCGCAAAT

CCTTCATTCCATGTTTAAGCTCCTACTAGGTTTAACCAAAAGGAGAAATGGAAATCTACC

TTTAACATGTGGGTCCCAGCAGAGCTTTTTAGCAAGAGTGCTCTTTCCAGAACCTCCCAG

ACCAGTCAAAACAAGAACACTGACATCATCCTTGAGAAGCTCAATCCTCAACCTGCTCAA

ATGCTCATCCATCCCCATACACTTGGGTTCTTCAGGAGCACCACTCAAACCCTGTATCTG

GCTCCCACTGAACTTAGCAAAATCTTGTTTTCCAAGAATGTTCAAAATCTCCTCAACCTT

CAAAAGAACCTCCATCAAGTTCCTCGCATTCTGAACCTGAACATCAAAGGACAAGTGCCT

TTGAAGTGACTGGTCCTCGGACTTAAGCTTCCCCTGGTAGTAAGGGAATGAAAGAAACTT

CCGGCGACTTAACTTTCTGGAGCACTTTCGTGCAAGTTCTTGTCCTTGACGTACGCGACT

CTGAAGCCTCTGGATCTCTTGTCTTGGTCGATCTAACTTGTCGTTGTATTCCATCATCTG

GTCAACCAGTGGTGCTATGTCGTTCAAGGTTTGTATGTTGGTTTCAAGTGTTGGCTTGAA

CTGTCGACCTTTGTTTATGGTTTCAATTACTGCTCGAAGAAGCTCTCCCATAACCGCTCC

AACGGCACCTCCACTGAACAGATCTGCCATACTCTAGTGATCGGATTGTCAGGTCAACCA

AGAGATTGATGGTGGTCAACGGCTTCACTACTAAAACAGAAGTTATTGTCTCAGCAAAAC

AAATGAAGTTGCCAATAGGATTATAGGAAGGATGGTAGCAACTAGCAATGT

>B05:9173668..9177669

TACTCTTTGAATTTTTAATGACTTAAAATATTCAATAATTGAATCAGAACTAAACTTTTC

AACAATTTCTTTCTCAATGTAAATAACCAAACTATCCGCAAGAAAGTCGTCCTCCATCTT

GTTTCTTAACCTTGTCTTTATTATTTTCATTGCTGAAAATGATTGCTCTGTGGTAGCTGT

AGAAACTGAAAGAGTTAATACAGACGAATCAATTTATCAATCAAGTAATACACTTTTGAT

TTTTTTGTTTCTGCTAAACATCTACACAGTTCATGAATAGTTGACAAATTCTTCATTTTT

AGATGCTGACGAACATCAAGAATAAAATGCTGAAGTTGAAAAGGCAAATTAATCTTCTCT

TATTCAATAAAGTTTTCGGGATAGTAGCTGTCAACAAGAATAGAAATCTTAGCAATGCCA

AAATTTTTATAAGCATCCTTAGACATGAGAGTAGAGCTCAAACTTAATAGATCCATTGCT

TGATTATTAAATTAAAGCATCTAAGCCAACTAAACTATTTTTTATTTTTTGAAAAACTAC

TACTAATTTTTTTATAAAAAATTTGGGGTAGCCATGCCACTGTCAGCAACGAACTCCATG

AAGCCAATTTAAGCTGATGTCTACTTTAGCTGTCTCTATCTTCAAATTGGGAGTTGAGGG

GACATTTTCCCATAATTGAGCCCTCTCCTCATCACATAACACAAATACTTGATGTTTTGC

ATTCTCAAAGTTCATGACTGAGTATGGCACCTCACTTAGCCCTGAACAACCCTTCATGTA

GAGTTTCTCAAGATTCTGCAGATCTCCAATGTCCTCTGGCAATTTGGATAGACTTACACA

GTCAGATATGTCGAGACAGCGCAGGTTACACAGTCGTCCAATCCCATCTGGCATCTCTTC

CAAATCGGAGCATGAACTTAGCCTTAGCACTTCCAAATTCTTCAGATTTGCTGTGTCTTT

AGGAAGTGCTGAAAGCTTGTGACAGTTGGTGATGCTAAGCTTCTTTAGTGGGGTAATGTT

GCATACCCCCTCTGGCAATTTCTCCAGATCTTTGCAATAGTCAATGCTCAACTCCACTAG

ATTTGGCATGGATTCTGAGATTGGGATAGAACTGTTCCCAAAAGCCTGGCTTGTGTTGCA

CATATGAAGCGATAGTTTTTGCAGACTCTTCAGTATGCATAAAGAAGGAACTGAAACTTT

CTCCAATCTGATTCTTCTCAAGTTAGACAAAGCACTCAGTATCTCAAAATCTTTTAGTCC

AGAAAGATGGAAGCCGTAATTTGTGACTATTAGAACTTTAAGTTTGTTCATTTTCTCCAT

GAACTGTGGCAGGCTGTACTGGCTAGATCGAATGTTTAAGACTATAACCTCGGCTTCATC

AGCTGTCATGTCGCACCAATCCGAAGCAAAATTCTCGTCTGCCCAAGCATCATATATGGC

ATCAAGAACACAAGATTATGCAAGATAAAGTAATTTAGATTTTAGAAGAGAGGAATTCAC

AAACCAGTTGATATAGACAATACTCTGGCAGCAACCTTTAGCTGCTTTTGTTCTATCAAT

CTTCCAGGTAAGAATGACAGTGACAAAAGGCGGCTGAATATTCCTTGCTGGTTTTGCCTT

ACCCACCATTCAGGATGATCATCTCCATTTAGCTCAATTATTAGTTTCTTTCTCTGTTCA

ATTTGTTCCTGCTCACTCTGATGGATTGCCAGCTTTCTGAGGAGATCATGTAGCAAGACA

AAGTGGTTATTGTAGTACATGTCTGTGTCCTTTGCAATTTTCCTGATCAATTACCATAGT

TTAGTCCAGCTATATTATGGAATCAATCTTTACTTGTCAATAAATCAAGCATTTTTAGAT

AACCAGCTACGAAAAGGATAAAGGATGATGTGTACTTTGTTACTATGAGCTTAATCAAGT

TCCTGGTGGTTAAATCATGGATGATGGTCATTGCTTTCCTTCCATTGTCATCAAAGTTAT

ACAGTTCTGCCCACATATCAATCAGGATTGCAACAGGGATCCTTTGGTCTTCAGGAAAAA

GGCCTAGATCCATGAAGCACTCCTTCTCACTAACTGAGAACTTATCTTCCAACATATCTA

AACTTTGTTGGAGCCGCGAAAGCAAATCTGTATTACCCGACTCCAAAATCGACTGCTTTT

CTAACCATTCTTTCATATCTTGCCACACCTCAAAAGGCTGCTGACACAGTGATCCACCAA

TCACTTCAAGTGCCAATGGTGAACCCTTACAACCTTTCACTATCTGAATTTCAGTGCAAC

AAAGCAAAAAATCAGTGAACAAGATCAAACAAAGAGCTTGAAAGAGTTACATGGAATACA

ATACACACATTCAAAGTAGAAAGAAGATAAAGCACCTCATTGACAAGATTATCATCTGGC

ATGTAGGAGCATTTACTATTAAGTTGAGCCGAGTGACGGAAAAGGGACACTGCATCATCA

TGATCAAGGGGTTCCAACGGCCATGGGGTGCCAAATCTTTGAAAAGCAACCCTTGAAGTC

ACCAAAATCTTATAATCTGGTATCTGAAATCTGAACTTGTCAACAAGTGCCTCTGAGCTC

TGCCAAACATCATCCAGGACTAATAGTATTGGGCTTCTCCCAACAAGCCTCAACAAAATT

CCCAATTTGTTGATGGCGTCTTCATCACTCTGAAACTCGGGCACCTTACATCCGCAGTGC

TCAAATAATGTCCTTACAATGTTCTTCAAGTTTGGTGTTTCTGAGAAGGTCACAAAGAAG

ATATTCCCCCCGAACTTGCCTAGCCAAACCAAGGTAAATTTCTATAATCAAGTAGTATCT

AGAAGTATTCCAGAATGCATGATATGCATGACCATTTCACTAGACTAAGAAGAGTAACGG

TATATAACTGAAAGCACCTACCTCAATTCTCAAGGCCACTGTAAACCTAATGTATCTGTT

AATGCATATGCATAGATAATTATGGTAATATGAATAAAAAAGGAGAAAGGAATTCTTTTC

ATATTGAAAAACATCAAAATTTTTACGAGGCTTTCCCAATTGAGATACTGTAACTAACAA

GAATGGACCACATTCCCGAAATAGCTCAAGTATTGAACTTGGTTTTGGTACGGTGACGGA

TACACATGACATTCTGGTATGCAAATAAATTGGTATGTGGTACTCGCAAATCCTTCATTC

CATGTTTAAGCTCCTACTAGGTTTAACCAAAAGGAGAAATGGAAATCTACCTTTAACATG

TGGGTCCCAGCAGAGCTTTTTAGCAAGAGTGCTCTTTCCAGAACCTCCCAGACCAGTCAA

AACAAGAACACTGACATCATCCTTGAGAAGCTCAATCCTCAACCTGCTCAAATGCTCATC

CATCCCCATACACTTGGGTTCTTCAGGAGCACCACTCAAACCCTGTATCTGGCTCCCACT

GAACTTAGCAAAATCTTGTTTTCCAAGAATGTTCAAAATCTCCTCAACCTTCAAAAGAAC

CTCCATCAAGTTCCTCGCATTCTGAACCTGAACATCAAAGGACAAGTGCCTTTGAAGTGA

CTGGTCCTCGGACTTAAGCTTCCCCTGGTAGTAAGGGAATGAAAGAAACTTCCGGCGACT

TAACTTTCTGGAGCACTTTCGTGCAAGTTCTTGTCCTTGACGTACGCGACTCTGAAGCCT

CTGGATCTCTTGTCTTGGTCGATCTAACTTGTCGTTGTATTCCATCATCTGGTCAACCAG

TGGTGCTATGTCGTTCAAGGTTTGTATGTTGGTTTCAAGTGTTGGCTTGAACTGTCGACC

TTTGTTTATGGTTTCAATTACTGCTCGAAGAAGCTCTCCCATAACCGCTCCAACGGCACC

TCCACTGAACAGATCTGCCATACTCTAGTGATCGGATTGTCAGGTCAACCAAGAGATTGA

TGGTGGTCAACGGCTTCACTACTAAAACAGAAGTTATTGTCTCAGCAAAACAAATGAAGT

TGCCAATAGGATTATAGGAAGGATGGTAGCAACTAGCAATGT

>A03:133776796..133780539

TCAATAAAAAGCGGGATTCAAATCCTGGTTCAGTACCACACGTATTCCCCACTTCTTCAC

ATCCACATAGTTGTGGGCATTAGCTTCGAATACAAAGCGGGATCCATGAAGATCATGCAG

CTTTCCATCCTGAATGAGTAGTTGGCGAGAAAGATAGAATAGGCAGAGGTGAACCAATTC

ACCCCTCACCTCCTCCTCCTTTTCATGCCAATAAACATAGTACACTAGGTTGCCTTTTCC

TGTATGGTCGTAAAAGTAGAGTTTAATGGAGAGGGATTGTTCAAATTGAACAAGAATGTG

GGCACAAAGGGCAATGCCAATCCAATTGTTGTCATTGGCATTCGGAAATGATTCTATCCA

CATTGAATTATCCGCCCCCTTATTCTGATCCTTAAACCACCTTGGAATTGGAACTCCACT

CCCTGGTATTACAATAGCAACTCTACCAGTGGATTCATTGTGTACCTGTAACCACAAAAA

CACAAACAACCCTAATCATTTGATAAACAAGAGCAGCAATAATGGCAACAACAATACGCA

CGCACCTTAATGTACTCTCTCATCCATGAACTACCCATTCCACTCAACCTTTCCTTCTCT

ACTATTTTCGGGCAGTTCAACATACTCAATTCTCCCCTTTTCACAAGTTTTGTTCGCTCT

GCCGCTGAATAGGATAATGGCAATGGGAACTCATCTACATACATTAGCCGTTTGCAGTTA

TCTAACACCAACACTCTCAGTCTGGGAAGCTTGTTTATGAAATCAGGTACTCTAACTATA

TTGTTTCCCTTGAAAAATAAGAACACTAAATAATGTAAGGATGATATGGCCTCAGGGATA

GTATGCAGATTACAATCACTTAGGTTAAGATAATTCACGCAGCTTAAGCGAGCCAGAGAA

AGCCTACACAGATGGAACACATGGTTGTATCTACTTTTAGAATAATTGAAGAAATGGAAT

GGTAACCTAAGCTTAAGCCACATATTTTAGTGAAGATATACCGAATACAGGGATGGGAAA

ACTCATTAAACTTGTGCACCCACTCAAATCTAACTGAACAAATTTTCTTAGGTCTCCAGT

TGATGGATGTATGTGCTTAAGCTTTCTACATCTTATAAGGAACAGCCGCTCAAGATTTGG

AGCCTGGCTAAGGTTTGGAGGCTTCACAAGATTTTCGGAGTCAAAGAGATTCAATTCCTT

CAAATTGTCCAGACACTGTTGCACAGTTAGAAAAAACAAACATCATTAATTACACTTTCA

CTTATTTGTTTGATAACTTCAAAATAAGTGAAGCTTATTTTATTTATTACTAGTACCATT

GTGCCATCGGATAGCTTACAACATGATGGAAAATACGTAAACGGATAATCATTCCACTCA

AGATATCGCAATTCGCTAGAAATGCTATTAAAACTTCCAGAAAAACTAACACTTTCTATT

CTGAGAAATTCAAGTCGTCTCATTCTTGATAATGTTTCTGCTCTTAATGTCATATTTCTT

TCTTCATCATATAATCCCCTCAATTGAACGGCCTTAACATCATCAATTACCTAAATACAA

AAAAGCCACAAAAATATTAGACTACTTCAATTACCTATTAACTCTATAAAATTGAAAATA

TTTTAAAACTGCATACCGTATTTCCAAACATAATACGTTGAAAATCCTCTTTGCACCATA

TCCTACTCCACTTCCATGGCTCATTTGGAGCACTTTGCCGAACTATTCTCATGCCTAACT

CTTGCAAACAGGTCATGCATTCTAATATAGAATCCATCAATTGTTATTAATGATTTATCG

ATGAGTACACTTATTCCAATCTTTGGATGAAGACCACGACAGTACAACATGTCTTCCACA

CGATAATCCATTTGGTGGTTAAAGAAACAAGCGATATCCAAAAATATTTCCTTTTCGAGG

GGTTCCAATCCATCAAAACTAAGTTGAAGCACATCTGCGATTTCCTTCTTTGGATTATTT

TTCAGTCTATCTAAGGCACTTCTCCATACAGAGATATCTCGACCGCGTAGATATGACCCC

AAACCTTTAACTGCCAAGGGAAGGCCTTGAGCATATTTAAGTGCAAGATCCGTCAACTTT

TTGTACTCTCTGCTAATCTCGTCACAACTCATAATTATATTATCATCAAAAGCTTTTTCA

CAAAACAATTGATGAGCTTCATCGTCATTCAAGAGTTGAACTTTGTATGTTTGATCTAGC

CCAAAAGAGTTCAAGACATGTTGATCTCTAGAAGTTACTACAATTCTGCTTCCTGGACAT

AGACAATCACGAGTCACGCCAAGCTTCTCTAACTGTTCAACTTCTTCGGCATTATCTAGA

ACAATCAGAGCCTTTTGACAACGTAGCATAGTTTTCATCAAACTCTTAGCTTCGTAATGA

TTGTATATATCTTGGATCTCTCCATTTGTAATTTGACAAACAAGTTGCTTTTGTAGAGAA

GATAGACCACCCGCATCACGAAAAACTTTGCTTATATCATCAACAAAACAATGAGCACCA

AATTTATGAAGGTTTCTACCATACACGACCCTAGCAAGTGTTGACTTTCCTATCCCACCC

ATGCCACAAACGCCTACAACCCGAACTTCATTATCGGAGTTGAAATCAATCAGGTTTTCC

AATTCCTCCACACGAGATTGCATCCCAACCAACCCATCCACACTTAACGAATTGCAACCT

AATTTGCTTCTCACCCTCTTAACAATTTTTTCAATCTCTCCATTCTCCTGCCTGAAAGAA

CAAAGAGAAAATGAACTTGTAAGCTCATTTTCTTATATAAGACAACCTAAAACAAAGAGA

AAACGAGAGTAATACAAGGTGATTTTGTTTAATCAACTTATTCATGTATAGATAGTAATG

GAATTTAGGAAAGATTACTTACTTATTTTGTATATCCCAACCAGAGAGATTGGCGACTTG

CAGCAGAGATTCCCTCCATTGTTGCACCATCTTTGAGTTATGTTTGAATCTTTCTTCATG

CTCAGCCAACGCTTTCCCATAGTCTCCGGTCTGTTTACGCACCTCAGACGGACTCACATC

GTAGAAAATAGGCAACAGATTTGGTTCTGTTATCGTATTGCTGCAATCAAGCATCTTAGC

GAGTTCCTGCAAGCACCATGAAGAGGTAGCATAGTTCTTAGAGAAGATGACAATGAGAAC

CTGAGATCCTTCAATTGCTTGCACCAGCTCAGTGGAGATGTGCTGTCCTTGCATCAGATT

CCTATCGTCTCTAAATGCAACTATTCCGTTTCTACGGAAAGCGGCAAAGAGATGATCAGC

GAAGGTGTTGCGTACGTCCTCACCTCTGAAGCTCACAAACACGTCATATTTGGGTCTTGT

GCTACTTGATGCCATGCTTTGGATGCTCCTGCACTCCATTGACGAAGTTGAAGTGAAGAT

TGGAAGAGACTGTGTGCAGAGAAGAAATATTCTGAGAAGTTGGATTGAAAGTCCTTTTTG

GAGGAAGGAAGCATCGTAGTAATTGAGATAAAGACTAATATTGTACCTTTAAAGGCCAAT

CAAGTCATATTCCCCTTGACTACTGTAACAAAACAAGTCTAGTTTATTTCATATTTTTTC

ATTATCACAATGGTATTAATTATTCTTGTGGGGTATAATGTTGAAAATACATTCTGCAAA

AGCAGAGGCTCACCAAAACTTTGTCTTCTTTGGGACCCAATGCAACTCTCTCTCTCTTGG

AATTTCAATTCCCGGAAATACCAT

>B04:132549922..132552596

TTATCGTAGAAATCTGTGAAGAGTTGTTACTTGATCTTTAACTTCTTGAAGTTGCCTCTC

TAAATCTGCGACATACTGTTGTGATGCACTGCTAGAACTTCCGCAATTTACAACTCCAAA

GATGTGCTTTGACTTACCAAAACCACGGGGACATATAGCATTATTAAAACCACGGACTCG

CTTACCATTATCATTTGGATGAGCTAAGACTTTTGAATGAATACCTTCAGTAGCAACACG

TTCTTGATCTTGAGACAAATATTCTGCTATTTCTTCCTACAAACAAATGCACATCGCATT

AGACTAGAGTATTCACATAATTAATGCATTAAAAAAAATATACAGCCATATAAAAAAAAT

TTTCAGACACACTCATGGCTAATTATTGTCCTTCCCTGCTTACATAACTCCCATCTTTCT

TTAGCAAAGTTGATACAATAACCTCGTTTCGATATATAGAGCTTCCCAATTTTTTCTCCT

AATACAATAAATAGAATAGTAAAGGTAATCACAAATTTAATTATAAACGATAATTTTATG

GAAATAGTATAATAGTACTAAGTACAATAAAAAAAATTATCATCTGAGTTGCTCTTCTAA

CATTGCTTTTACTTCCACCGACATGTGAAACTATAAGTTTCTCACGATTTCTAGCGTTTT

GCAAATATTGTTTTTATGATTTTCAAAAACAAAAAATTAGTAAATAAATATTACCAGATA

CTCATAGTCTTAATAAAAAAAAACACAAAAATATAGAATAAGTATTTATTTTTGTCTTAG

AGTCTTTATAATGATCCACAAAAACAGTCCATTTAACAAGTGGTATGTCTGAAGAATTTG

CAGTCAAAATTTTTGTCTTTCTTTTATTAGGATAGAAGTATAACCCTCGTAAATTATATT

TATGGGTAAAATTCATGTGAATTTTTTATTAAGCAAAACACTATATTCATAAACAAGTTA

ATGTGTTAGGTTTAAAAGTTAGATGTGTTTTTATGAGATTTTTGTCTCAATAAAGTATAC

ACAGGCTTACACACACCTGAGGATCAAGTTTGATTCCAATTACTTTAGTTTTTGTGAAAT

TGATGTTTTAATTTTATATATGTAGTGAAAGCTTTAGAAATAATAATATTTTTTAATTTT

AAATTGATTATTGTCAAACTAATCAAGGACATACATTACCATGAAGATGAGTACTTTAAT

TGATAATTAATACTAATCGCATACTTTTTAAATAGAAGAAAAAAATTATTACATAAAAAT

TAAAGATGGTGACATATATAACACGTACACATAAGGCAAATCAGCTTTTGGCAAACCTAT

CCTGCAAGACCAACAACTTAATCATATTGTACTAGTAGAACTAGCCAAATTAAATTCTTA

TACATATATTGGACTGGTAATGGTAGGGTAGAGTTTAGACTCTACTTTAATCCTATTCGC

GAGTTGAAAACTTTTATAAAACTATACCCTACCCTACCCACGGGTTTAGAATCTCTCAAC

TCTAAGTTTACCGCACTCTAAAATTATAAATCCTACCCTACCCGATTTTACTCGCAGAAA

CAAAAAAAAATTCAAATAAATATAAAATTCAACCATTCCAAATTTAATACATATTAATAA

AATAGAAAATAAAAAACTAAGTTCAAATTAAAATTAAAAACAATAAAATCTTAAAGAAAT

TCAACATAAAATTACAAATAATATGATCATCAACTAACTTAGTGATTATTTCAAGTTTTT

ATAAGAAGAAGATTGTGAATAAAAGACTCACTTTTTTTCACTACATACATAACTTTTACA

TATATATTATATAATATATTAAGGATGCAGGTAGGGTAGAATAGAATATACACTAAACTC

GTACCCTACCCTACCCACAGACAAATTTACACCCTATTCTACCCTACCCGCAGCGGGCGG

GTACACAACCCTACCCAAATGGATTGGTCCGAGTTGAGTACCTGCGGATAGGATATATAT

TGCCAGCCCTATACATATATTATATAACCTGTATGCAAGGGAGATGAAGAGTTTAATTAA

TTTGGTTATTTAAGTAACGAAAATATTTAGTAATTAAAATAAATTAATTAAAAACAGTTA

AAAATTATCAAATATTAAATAAAATAAATTCTGACTATTTTCTTTCTTTAACATTACTTG

GTAATAGAAATGTTTGGTAATATATAAGGACGTTGAGTTGGAAATTTGTGTGAAGGAAAA

TAATAACAACTAATAAGAATGCCTTTTTTAATGTCGTGACATCTGATTCTGGCGCAGCCT

AGTAAAGTAGTTCCACCCCAAACAACTTTAACATAGGGAACACAGCTATAATTCTTCTTA

TCAGTACAAGAGTTGGATTTGTAATCATAATTTTTTTTCGTTTCTACCCAAAGAGCCACA

GCATCTGCTCCAGTAATAAGTCGTGAATTTTGTTCAACAAGTCGGCTAAATCTACCACCA

GTACCACCCTTTCCTAATTTACAACTTTCAACGTGTTTATTCACGAATGTCCTAGCATGA

TCTTCCAACTTTATGTCCCACTTCAATGGTTGAACTCCAACACTTGCACGTGCATCGTTG

TGAACTTTAAGGTAGTCTTTTGGATAGTTTTGAGCCATTAAAAATAATGGAACTACACTT

ATGATGAAACTTAATACCGCTACCCAAATCTTCAT
